# Lessons learned from annotation of VAERS reports on adverse events following influenza vaccination and related to Guillain-Barré syndrome

**DOI:** 10.1186/s12911-023-02374-2

**Published:** 2024-01-05

**Authors:** Madhuri Sankaranarayanapillai, Su Wang, Hangyu Ji, Hsing-Yi Song, Cui Tao

**Affiliations:** 1 McWilliams School of Biomedical Informatics, The University of Texas Health Science Center, Houston, TX USA; 2 Department of AI and Informatics, Mayo Clinic, Jacksonville, FL USA

**Keywords:** Vaccine adverse events, Influenza vaccine, VAERS, Guillain-Barré syndrome, MedDRA

## Abstract

**Background:**

Vaccine Adverse Events ReportingSystem (VAERS) is a promising resource of tracking adverse events following immunization. Medical Dictionary for Regulatory Activities (MedDRA) terminology used for coding adverse events in VAERS reports has several limitations. We focus on developing an automated system for semantic extraction of adverse events following vaccination and their temporal relationships for a better understanding of VAERS data and its integration into other applications. The aim of the present studyis to summarize the lessons learned during the initial phase of this project in annotating adverse events following influenza vaccination and related to Guillain-Barré syndrome (GBS). We emphasize on identifying the limitations of VAERS and MedDRA.

**Results:**

We collected 282 VAERS reports documented between 1990 and 2016 and shortlisted those with at least 1,100 characters in the report. We used a subset of 50 reports for the preliminary investigation and annotated all adverse events following influenza vaccination by mapping to representative MedDRA terms. Associated time expressions were annotated when available. We used 16 System Organ Class (SOC) level MedDRA terms to map GBS related adverse events and expanded some SOC terms to Lowest Level Terms (LLT) for granular representation. We annotated three broad categories of events such as problems, clinical investigations, and treatments/procedures. The inter-annotator agreement of events achieved was 86%. Incomplete reports, typographical errors, lack of clarity and coherence, repeated texts, unavailability of associated temporal information, difficulty to interpret due to incorrect grammar, use of generalized terms to describe adverse events / symptoms, uncommon abbreviations, difficulty annotating multiple events with a conjunction / common phrase, irrelevant historical events and coexisting events were some of the challenges encountered. Some of the limitations we noted are in agreement with previous reports.

**Conclusions:**

We reported the challenges encountered and lessons learned during annotation of adverse events in VAERS reports following influenza vaccination and related to GBS. Though the challenges may be due to the inevitable limitations of public reporting systems and widely reported limitations of MedDRA, we emphasize the need to understand these limitations and extraction of other supportive information for a better understanding of adverse events following vaccination.

## Background

An adverse event is defined as “an unintended injury or complication which results in disability, death or prolonged hospital stay and is caused by health care management” [1]. Identifying new adverse events and monitoring them to understand the underlying reasons are essential to prevent these adverse events in future encounters [2]. In this regard, adverse events reporting systems are an essential part of surveillance and pharmacovigilance. Vaccine Adverse Events Reporting System (VAERS) was established in 1990 by the Food and Drug Administration (FDA) and Centers for Disease Control and Prevention (CDC) to collect reports on adverse events caused by vaccines licensed in the U.S. VAERS serves as a passive reporting system that collects reports filed by individuals based on their experiences. All VAERS reports are coded before they are entered into the database. Adverse events in VAERS reports were coded using FDA's Coding Symbols for a Thesaurus of Adverse Reaction Terms (COSTART) until January 2007. VAERS coding system changed on January 17, 2007, to an international medical terminology called the Medical Dictionary for Regulatory Activities (MedDRA) and all COSTART codes were converted into MedDRA terms.

VAERS reports may be filed by any individual including patients, family members and healthcare providers on a voluntary basis [3]. VAERS is especially useful in identifying uncommon and unexpected trends of adverse events that are indicators of potential problems with a vaccine [3]. However, several limitations exist in using VAERS reports due to the passive nature of this surveillance system such as incompleteness, errors in reporting and quality of the reports, etc. Retrieval of meaningful information from VAERS reports about the adverse events and their time course following the suspected cause (vaccine) is essential in predicting the potential occurrences of these serious adverse events, to aid in vaccine safety evaluation, to provide recommendations and regulatory action for preventive care and improve vaccine safety measures [4].

More strictly speaking, the causal relationships between a reported event and the vaccine in VAERS are not usually guaranteed or verified. Adverse events following immunization (AEFI) are temporally associated events that are observed to occur following the administration of a vaccine which may or may not have caused the adverse event [4, 5]. According to the definition of the Council for International Organizations of Medical Sciences (CIOMS), AEFI may or may not have a causal relationship with a vaccine usage [6]. Finding a causal relationship between an adverse event and a vaccine requires strong scientific evidence that has to be supported by the establishment of a temporal relationship in addition to other factors [7]. This underlines the importance of extracting meaningful information from the adverse events along with the associated temporal events in VAERS reports.

Extensive studies were conducted on the use of MedDRA terminology and VAERS data and to find better ways of meaningful representation of both structured and unstructured forms of data in VAERS. An Ontology of Adverse Events (OAE) has been developed to provide logical definitions and classifications of various adverse events following medical interventions [8]. Chute et al., used Time Event Ontology (TEO), OAE and Vaccine Ontology (VO) for semantic representation of VAERS data and developed Temporal Information Modeling, Extraction, and Reasoning (TIMER) framework for extraction and reasoning of temporal information in VAERS reports automatically [9]. Chen et al., demonstrated the potential of a statistical approach based on a random effects model to detect the heterogeneity of vaccine and adverse event reporting rates over time in VAERS database to identify safety signals [10]. He et al., identified statistically significant vaccine adverse events associated with monovalent and combination vaccines against Hepatitis A and B and a comparative analysis of these adverse events and vaccine vaccine interactions (VVI) showed potential risks of adverse reactions following two monovalent vaccines and one combination vaccine [2]. The authors also compared the use of OAE and MedDRA for the classification of adverse events and noted the advantages of OAE and highlighted several limitations of MedDRA [2]. In our earlier study, we conducted a comprehensive analysis of adverse events in VAERS database using a combination of statistical methods and terminology grouping and studied Trivalent Influenza Vaccine at System Organ Class (SOC) level of MedDRA terms [11].

While several studies discussed the limitations of MedDRA in representing the adverse events in VAERS systems, we focus on exploring these challenges of using MedDRA in the process of extracting useful information from VAERS reports through temporal relationships of adverse events. Our use case in the present study is the vaccine-adverse event pair of influenza vaccine and Guillain-Barré syndrome (GBS).

GBS is a rare but severe autoimmune disorder in which the body’s immune system attacks a part of the peripheral nervous system and is often preceded by a viral or bacterial infection [12]. Administration of vaccines and surgery are known to trigger GBS [12]. The risk of adverse events such as GBS does not outweigh the benefits of influenza vaccination [13] and controversies are reported about the risk of GBS following influenza vaccination [14]. However, many studies have highlighted GBS as a major concern of vaccine safety since the major outbreak of GBS following the US National influenza immunization programme against Swine Flu subtype A/NJ/76 in 1976 [15, 16]. Hence, we primarily focused on GBS related post-vaccination adverse events that are reported to VAERS.

## Results and discussion

**Fig. 1:**
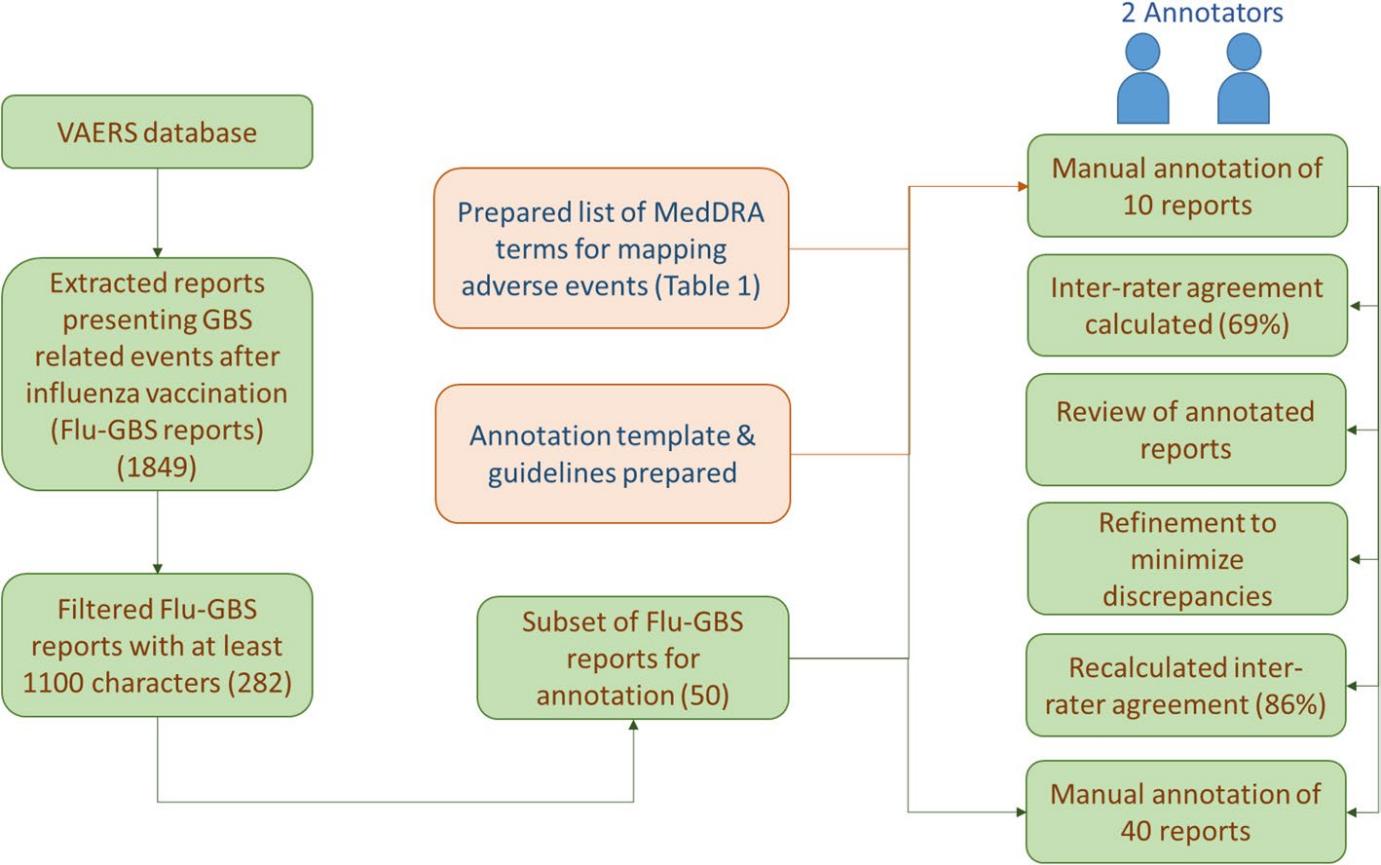
Workflow of the annotation process of VAERS reports

**Fig. 2:**
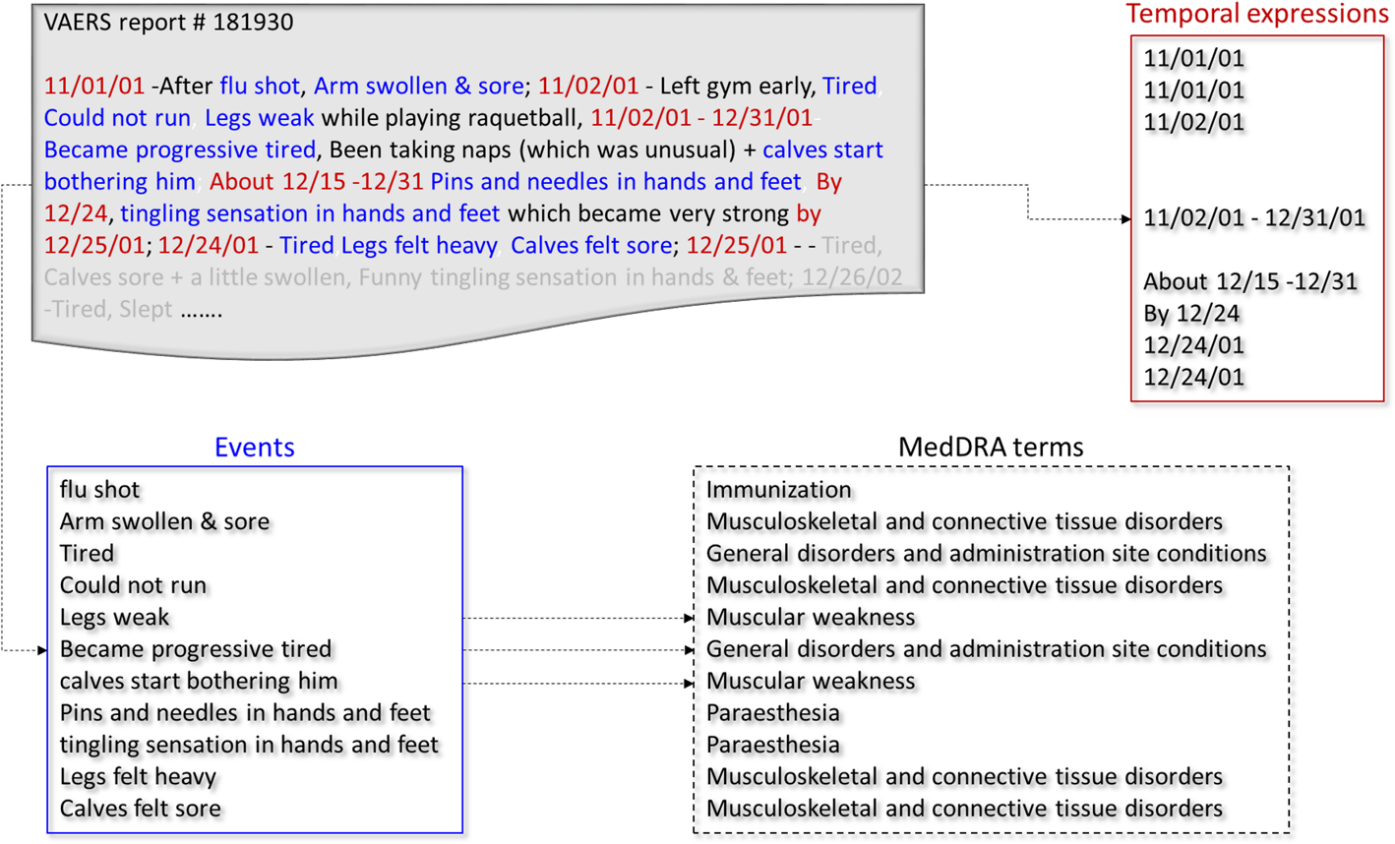
An example report illustrating selection of adverse events, time expressions and mapping of adverse events to MedDRA terms

Figure 1represents the complete annotation process of VAERS reports. Our initial search in the VAERS database for serious adverse events of interest including GBS as one of the adverse events resulted in 2634 reports. Further search focused primarily on serious adverse event reports indicating the manifestation of typical symptoms related to GBS after administration of influenza vaccinations (listed in Section A) resulted in 1849 reports. A threshold of 1100 characters set as report length limited our selection to a subset of 282 VAERS reports. For our preliminary investigation, we randomly selected a subset of 50 VAERS reports from the selected 282 cases reporting onset of GBS after influenza vaccine administration. For manual annotation, all adverse events reported after influenza vaccine administration were selected and mapped to MedDRA terms (listed in Table2). Table2also shows the number of occurrence of events mapped to each MedDRA term. The time expressions associated with the adverse events were also noted when available. An example report illustrating the selection of adverse event phrases, time expressions and mapping of adverse events to MedDRA terms is shown in Fig. 2.

For the first phase, two independent annotators with domain expertise annotated a randomly selected initial set of 10 reports. The inter-annotator agreement of 69% was achieved as estimated by Kappa coefficient. In addition to adverse events, the two annotators also annotated time expressions (timestamp, duration, frequency, etc.) associated with the events in reports. In order to handle inconsistencies in the reports and minimize discrepancies between annotators, both annotators reviewed the annotation results carefully, refined the annotation rules further and revised the annotations based on new guidelines. This resulted in an improvement of inter-annotator agreement for annotation of events as Kappa coefficient increased from 69 to 86%. As a next step, the two annotators annotated the rest of 40 reports using MedDRA terms as before. The annotation results of all 50 reports were pooled and thoroughly reviewed to minimize discrepancies between the two annotators.

The manual annotation process of the narrative text in VAERS reports was not easy and somewhat challenging due to the inherent limitations of VAERS reports. Here we provided a comprehensive overview of some of these challenges. Specific examples of text segments (in*italic*text) from VAERS reports are included to illustrate the commonly encountered problems in the annotation process (**in underlined bold text**).

1. Many VAERS reports are incomplete and contained several typographical errors.

Example:

*Could not sleep—very uncomfortable all ****nite*** *and morning (ID# 181930).*

*On 02/10/2000, he was discharged with a final diagnosis of Guillain–Barre syndrome to a* ***rehabiitation*** *facility for physical therapy. (ID# 185520)*

*… after she developed an elevated BUN and creatininie* ***who had a history of was felt to be*** *secondary to the immunoglobulin (ID # 164505)*

*Calves still sore & ****slighty*** *swollen, Not walking very well (ID # 181930)*

*when I went to get out of bed my legs could barel ****hold be up****. (ID# 454439)*

2) In many cases, reports were vague, lacked clarity and coherence.

Example:

*I got up from my desk and on my way back,* ***my right leg gave out from under me****. (ID# 458909).*

*Pt later reported ****band-like numbness*** *around the abdomen and increasing difficulty walking. (ID# 304964)*

*On an unknown date, ****the outcome of the cataplexy was not recovered/not resolved*** *and* ***the outcome of the unable to walk, Guillain Barre syndrome and viral infection were unknown****. (ID# 594878).*

*Couldn't walk up steps describes ****legs feeling like jelly****. (ID# 339281)*

3) Some reports contained repeated texts (example ID# 185520, 192326) and it was challenging to follow the order of events with unclear texts.
4) Many of the reported adverse events did not have a well-defined time stamp or time expression associated with it. (example ID# 602409, 392187).
5) The available time expressions were incomplete and lack important details. (example ID# 183412, 178909).

Example:

*Initial symptoms ****began in late September / early October*** *and progressively gotten extremely worse* ***by end of October / early November*** *(ID# 531610).*

***On an unknown date****, the patient received FLULAVAL. (ID# 594878)*

6) It was difficult to interpret the exact message conveyed from the reports that has incorrect grammar and unclear texts, as given in the following example:

*On an unknown date, ****the outcome of the cataplexy was not recovered****/not resolved and ****the outcome of the unable to walk, Guillain Barre syndrome and viral infection were unknown****. (ID#594878).*

*12/28/01—11:45 AM appt with family Dr, Could hardly walk, Tingling very bad in hands and foot, Dr did physical and took x-rays, All seems normal, ****Dr you was baffled****; (ID# 181930)*

7) Some of the adverse events / symptoms reported were described by generalized terms and it was challenging to annotate them with a more appropriate / specific MedDRA term.

Example:

*Calves****still*** *sore & slighty swollen, ****Not walking very well*** *…* ***Calves very swallow****(ID # 181930).*

*The patient's ****condition continued to decline****. (ID # 168255)*

8) We found that many events in the narrative text in VAERS were described as abbreviations and some of the abbreviations were either unfamiliar or they are medical abbreviations that required domain expertise for interpretation. Annotation of these abbreviations was more time consuming than explicit events.

Example:

*he had a history of hypertension, left* ***cva*** *and a ****fib***.*(ID # 183412).*

***RTC*** *3/17/09 improved, started on steroid (ID # 341144)*

9) It was difficult to annotate multiple events with a conjunction or a common phrase as in the example below when it was necessary to annotate each independent event. The two events in the examples below were annotated using the MedDRA terms Physiotherapy and Occupational therapy respectively.

Example:

***Physical and occupational therapy*** *(ID # 374614).*

*During the 12 days in the hospital I received 5 plasmapheresis treatments with ****physical and occupational therapy*** *sessions. (ID # 480317)*

10) Some historical events either directly related to the patient or to the patient’s family are also found in VAERS reports. We noted that it was important to identify historical events and exclude them from annotation as these events are not directly relevant to our study on influenza vaccine adverse events. Identifying and excluding irrelevant historical events from narrative text of VAERS reports was challenging and time consuming.

Example:

***The patient's past medical history*** *included an* ***umbilical hernia*** *which was repaired in Sep-2009. (ID # 523530).*

*Also states that he had a ****history of hypertension, left cva and a fib***. *(ID # 183412)*

*Pt had a ****Pen allergy in childhood*** *resulting in* ***respiratory arrest****. (ID # 374614)*

*strong ****family history of diabetes mellitus*** *and* ***stroke****. (ID # 187767)*

*One ****family member*** *who died of a ****cerebral aneurysm****. ****Mother has factor 5 Leiden****. (ID # 298477)*

11) Though some adverse events were reported as coexisting events, they had to be annotated as independent events using appropriate MedDRA terms.

Example:

*Patient presents to ED on 10/30/13 with a chief complaint of* ***bilateral lower extremity weakness*** *with* ***muscle and joint pain*** *(ID # 512865).*

*The patient stated that he had* ***a mild URI*** *with* ***fever and chills****…(ID # 187767)*

12) Some narratives were ambiguous to be treated as either events that had happened or plans that may have not happened.

Example:

*Visits ****will**** be two times per week* ***until stronger*** *and then three times per week ****until goals met****. (ID # 549,426).*

*Hopefully by then my legs should be better and* ***if not*** *I* ***will*** *have to continue with disability and doing fitness training. (ID # 458,909)*

13) It was difficult to annotate the temporal expressions used to represent the time frequency of a series of independent events such as treatments / procedures.

Example:

*1/2/07 Received medical records from hospital which reveal patient admitted ****3 times: 10/13/06–10/19/06****. (ID # 267,701).*

*Follow-up visit with neurologist* ***every two weeks*** *and recovering from nerve damage at home* ***until January 2, 2009****. (ID # 334,072)*

In addition to these challenges of VAERS reports, we notedseveral issues in use of MedDRA mapping to code adverse events in VAERS. According to VAERS data user guide, each semi-annual update of MedDRA coding system adds new terms and deletes some old terms. Due to these changes, similar adverse events reported at different times may be represented by different MedDRA terms, depending on the MedDRA version in effect at the time of release [17]. Further, the list of Symptoms in VAERS is not free from duplicates [17]. Furthermore, the lack of term definitions and hierarchical structure in MedDRA as reported earlier [2, 8] add more limitations to the already complex nature of VAERS reports.

The narrative reports in VAERS are highly valuable sources for detecting potential adverse events of vaccination. However, the descriptions of many adverse events in these reports are not standardized or even vague as mentioned above. Hence, substantial domain expertise is required for manual review of a large number of spontaneous descriptions of adverse events before mapping them to MedDRA, making the manual annotation of narrative reports an expensive task.

## Conclusions

For automated retrieval of adverse events from narrative reports and mapping them to MedDRA, we focus on developing a corpus of adverse events following influenza vaccination and related to GBS. We suggest that improving VAERS system with (1) a more structured interface for users to select pre-defined terms for better representation of adverse events, (2) providing more detailed temporal information of the reported events and (3) development of an automatic system to identify adverse events relevant to a specific vaccine may improve the use of VAERS reports for research purposes and to automate meaningful information retrieval. Further, the use of better knowledge representations such as OAE [8] may help overcome the limitations of MedDRA mapping to adverse events.

In the present study, we reported the challenges encountered and lessons learned during the annotation of adverse events in VAERS reports, related to GBS followed by Influenza vaccine. Though these challenges are mostly due to the inevitable but inherently complex nature of narrative text in a public reporting system such as VAERS and widely reported limitations of MedDRA terms encoding the adverse events reported in VAERS, it is important to recognize and understand these challenges for meaningful extraction of the adverse events from the narrative text of VAERS reports and to identify the temporal relationship between the events, to perceive and understand the sequence of the adverse events following vaccination. Further, we noted that it is important to extract other relevant details such as severity of adverse events, negation, improvements, historical events (patient / family history) etc., in addition to identifying adverse events following vaccination to provide a comprehensive understanding of the adverse events following vaccination in the course of time. We believe that the lessons learned in this study provided a better guidance for our work in progress on temporal annotation of adverse events following influenza vaccination and related to GBS.

## Methods

VAERS data contains both narrative text as well as structured data. Although the structured data in VAERS reports have been used by many statistical analyses, the narrative text usually provides valuable additional data especially temporal information such as timestamps, duration, frequency, etc., about post-vaccination events. Hence extraction of the temporal information available in the narrative text may provide useful insights into the sequence of events following vaccination and assessment of significant clinical features such as causality, exposure and vaccine safety [7]. However, the extraction of such valuable information hidden within the unstructured narratives is a challenging task [9]. Based on our prior studies, we are currently focusing on the development of an automated system for semantic extraction of post-vaccination adverse events and their temporal relationships for a better understanding and integration of VAERS data. In the present study, we summarize the lessons learned in the preliminary phase of this project during the annotation of VAERS reports with emphasis on the limitations of VAERS and MedDRA. To the best of our knowledge, our study is unique and first of its kind attempting to extract temporal relation between influenza vaccine and GBS.

### Collection of VAERS reports

We searched VAERS database and retrieved reports that documented serious adverse eventssuch as death, life-threatening illness, hospitalization, prolonged hospitalization, permanent disability, etc. and collected during the time period 1990—2016. Among these reports, we selected a set of reports that specified GBS as one of the adverse events following any of the influenza vaccination including FLU3, FLU4, FLUA3, FLUC3, FLUC4, FLU(H1N1), FLUN3, FLUN4, FLUN(H1N1), FLUR3, FLUR4, FLUX, FLUX (H1N1) and H5N1. To ensure adequate information to identify GBS related adverse events following influenza vaccination, we set a threshold of 1100 for the number of characters as report length and limited our search to only those reports that fulfilled this selection criterion and used the resultant subset of VAERS reports for further processing.

### Medical Dictionary for Drug Regulatory Activities (MedDRA) terms for mapping

We identified the adverse events encountered commonly in patients with GBS from available literature [18, 19] and from VAERS reports. They are listed in Table1. GBS related adverse events found in VAERS reports were mapped to MedDRA terms, specifically, 16 SOC terms under the MedDRA hierarchy. Some of the SOC terms that encompass more frequently reported GBS- related adverse events were further expanded to Lowest Level Terms (LLT) for more granular representation and mapping of the adverse events in VAERS reports. Table2shows the complete list of MedDRA terms used for mapping the adverse events.

**Table 1:** Frequently encountered adverse events in GBS patients

Pulmonary disease and symptom	• Respiratory illness • Upper respiratory infectious symptoms • Respiratory insufficiency • Pneumonia • Pulmonary embolism
GI disease and symptom	• Diarrhea • Gastrointestinal bleeding
Neurologic symptom	• Numbness • Paresthesia • Weakness • Pain in the limbs • Weakness of the limbs • Weakness progresses • Generalized hyporeflexia • Generalized areflexia • Unable to walk
Infection	• Campylobacter jejuni infection • CMV infection • EB virus infection • Influenza virus infection • VZV infection • HIV infection • Mycoplasma pneumoniae infection
Clinical intervention	• Surgery • Vaccination
Test	• Spinal tap (lumbar puncture) • Electromyography • Nerve conduction studies
Other	• Sepsis • Hodgkin's lymphoma

**Table 2:** List of MedDRA terms used for annotation

System Organ Class (SOC)	Lowest Level Terms (LLT)	Number of occurrences
Investigations	Other investigations	205
	Computerised tomogram	8
	Electromyogram	8
	Physical examination	6
	Anti-ganglioside antibody	0
	Albumin CSF abnormal	0
	Oxygen saturation	0
Nervous system disorders	Other Nervous system disorders	180
	Muscular weakness	146
	Guillain-Barré Syndrome	119
	Hypoaesthesia	93
	Paraesthesia	58
	Difficulty in walking	43
	Areflexia	18
	Headache	14
	Ataxia	10
	Dizziness	4
	Muscle spasm	3
Surgical and medical procedures	Immunization	134
	Other surgical and medical procedures	111
	Hospitalisation	89
	Rehabilitation therapy	41
	Emergency care	39
	Physiotherapy	32
	Plasmapheresis	24
	Intensive care	20
	Catheter placement	7
	Intubation	7
	Tracheostomy	4
	Mechanical ventilation	2
	Occupational therapy	1
	Cardiac pacemaker insertion	0
Social circumstances	Other social circumstances	26
	Impaired work abilities	11
	Foreign travel	0
General disorders and administration site conditions		157
Musculoskeletal and connective tissue disorders		104
Respiratory, thoracic and mediastinal disorders		92
Gastrointestinal disorders		59
Infections and infestations		35
Renal and urinary disorders		33
Other organ system disorders		32
Cardiac disorders		18
Eye disorders		12
Blood and lymphatic system disorders		9
Psychiatric disorders		9
Endocrine disorders		5

### Annotation guidelines

We followed the guidelines below for annotating events and temporal expressions in VAERS reports.

#### General guidelines

1. We annotated time expressions, time stamps, durations and events that may or may not have an associated time or temporal relation.
2. We annotated only events that already occurred.
3. When annotating time, prepositions in the text such as “by”, “on”, “in”, “about”, “approximately”, “for”, “within”, etc. were included. Example:for 3 days*, *On 12/28/00
4. When annotating events related to Flu-GBS, prepositions were not included unless they are modifiers of the event. For example, in the text “*severe pain*”, the preposition “*severe*” is a modifier of the event “*pain*”. Therefore, it was included. “*had physical therapy*” was annotated as “physical therapy”.
5. We annotated events in discontinuous segments, independently. For example, the text segment “*did physical & occupational therapy*” was annotated as “physical” and “occupational therapy”.
6. Generalized events were not included for annotation. For example, “*The patient’s condition continued to decline”*
7. Negated events were not included for annotation. Example: “*no peripheral edema”*
8. We included past historical events of the patient for annotation. Example: *PAST MEDICAL HISTORY: *Fracture of right ankle in 4/00, … history of bunionectomy in 1986

#### Annotation of events

We classified the events into three broad categories such as problems, clinical investigations, and treatments/procedures. Although our primary focus is on GBS related adverse events (problems) following influenza vaccination, we included clinical investigations as well as treatments/procedures in the “events” category because annotating these closely related events may provide a more comprehensive understanding of the complete sequence of adverse events following vaccination. Table3shows examples of VAERS text annotation of events under each of the three categories described below.

**Table 3:** Annotation examples of events from VAERS text

*Description of events*	*Mapped to MedDRA term (SOC / LLT or SOC)*	*Annotated event (in bold text) examples*
Medical problems: Phrases / words that specify a disease, syndrome, symptom or sign	Nervous system disorders (SOC) / Guillain-Barré Syndrome (LLT)	“*suffering from* ***Guillain–Barre Syndrome****”*
	Nervous system disorders (SOC term) / Ataxia (LLT)	*“pt was admitted into hospital for* ***loss of mobility****”*
	Gastrointestinal disorders (SOC term)	*“developed* ***vomiting*** *and* ***diarrhea****”*
		*“symptoms of* ***heart burn*** *suspected of* ***gastroesophageal reflux disease****”*
Non-medical problems that impact social life	Social circumstances	*“Pt's current condition is* ***confinement to a wheel chair****”*
		*“truncal weakness with* ***inability to support himself****”*
Clinical investigations	Investigations	*“****MRI****,****CT scan*** *was suggestive of spinal tumor and fracture”*
		*“Had repeat* ***EMG****/****NCS*** *done as outpatient”*
Treatments / procedures	Surgical and medical procedures	*“the pt received 6 courses of plasmaphoresis”*
		*“Patient was hospitalised for evaluation, and received* ***tracheotomy*** *as result of difficulties”*
		*“10/15/14 referral to* ***physical therapy*** *due to weakness and short endurance”*

##### Problems

These are phrases/words that contain observations, symptoms, and diagnoses reported by the patient/reporter (family/friend/physician/unknown) related to the patient’s body or mind and are considered as unusual/abnormal / caused by a disease. These medical problems are grouped into the SOC terms in the MedDRA hierarchy such as nervous system disorders, gastrointestinal disorders, etc. There are also non-medical problems that impact social life. Such problems belong to the group under the SOC term ‘Social circumstances’.

Phrases/words that specify a disease, syndrome, symptom or sign related to the nervous system but not covered by the sub-entities (LLT terms) listed under ‘Nervous system disorders’ (SOC term) were annotated as Other nervous system disorders.

##### Clinical investigations

These terms include names of tests, exams and other techniques of clinical investigation.

##### Treatments/procedures

These terms relate to treatment methods, medical and surgical procedures carried out on the patient.

#### Annotation of temporal expressions

All temporal expressions associated with the events annotatedas described in Section (b) were identified and annotated as time expression. Temporal expressions may be time, day, date, year and / or duration of the reported event. Some examples of reported text containing temporal expression (shown as underlined text) are given below.

##### Time

Total face was paralyzedas of 8:00AM.

##### Day

He also had nausea and vomiting that weekend, and was unable to keep any food downuntil Monday.

##### Date

13th, pt admitted to Hospitaluntil the 17th.

##### Year

In March 2012, an unknown time after receiving FLULAVAL, the patient experienced cataplexy.

##### Duration

Received physical therapy at homefor 4 to 6 weeks.

## Data Availability

VAERS reports used in this study are publicly available here:https://vaers.hhs.gov/data.html

## Abbreviations

AEFI: Adverse events following immunization
CDC: Centers for Disease Control and Prevention
COSTART: Coding Symbols for a Thesaurus of Adverse Reaction Terms
FDA: Food and Drug Administration
GBS: Guillain-Barré syndrome
LLT: Lowest Level Terms
MedDRA: Medical Dictionary for Regulatory Activities
OAE: Ontology of Adverse Events
SOC: System Organ Class
TEO: Time Event Ontology
TIMER: Temporal Information Modeling, Extraction, and Reasoning
VAERS: Vaccine Adverse Events Reporting System
VO: Vaccine Ontology
VVI: Vaccine vaccine interactions

## References

[1] Wilson RM, Runciman WB, Gibberd RW, Harrison BT, Newby L, Hamilton JD (1995). The quality in Australian Health care study. Med J Aust.

[2] Xie J, Zhao L, Zhou S, He Y (2016). Statistical and ontological analysis of adverse events associated with monovalent and combination vaccines against hepatitis A and B diseases. Sci Rep.

[3] About VAERS;https://vaers.hhs.gov/about.html. Accessed 9 Apr 2019.

[4] Shimabukuro TT, Nguyen M, Martin D, DeStefano F (2015). Safety monitoring in the Vaccine Adverse Event Reporting System (VAERS). Vaccine.

[5] Centers for Disease Control and Prevention Understanding the Vaccine Adverse Event Reporting System (VAERS). 2013.http://www.cdc.gov/vaccines/hcp/patient-ed/conversations/downloads/vacsafe-vaers-color-office.pdf.

[6] Council for International Organizations of Medical Sciences (CIOMS) (2012). Definition and Application of Terms for Vaccine Pharmacovigilance: Report of CIOMS/WHO Working Group on Vaccine Pharmacovigilance.

[7] Loughlin AM, Marchant CD, Adams W, Barnett E, Baxter R, Black S (2012). Causality assessment of adverse events reported to the Vaccine Adverse Event Reporting System (VAERS). Vaccine.

[8] He Y, Sarntivijai S, Lin Y, Xiang Z, Guo A, Zhang S (2014). OAE: The Ontology of Adverse Events. J Biomed Semant.

[9] Tao C, He Y, Yang H, Poland GA, Chute CG (2012). Ontology-based time information representation of vaccine adverse events in VAERS for temporal analysis. J Biomed Semant.

[10] Cai Y, Du J, Huang J, Ellenberg SS, Hennessy S, Tao C (2017). A signal detection method for temporal variation of adverse effect with vaccine adverse event reporting system data. BMC Med Inform Decis Mak.

[11] Du J, Cai Y, Chen Y, Tao C (2016). Trivalent influenza vaccine adverse symptoms analysis based on MedDRA terminology using VAERS data in 2011. J Biomed Semant.

[12] Guillain–Barré syndrome; Available from:http://www.who.int/news-room/fact-sheets/detail/guillain-barr%C3%A9-syndrome. Accessed 9 Apr 2019.

[13] Principi N, Esposito S. Vaccine-preventable diseases, vaccines and Guillain-Barre' syndrome. Vaccine. 2019;37(37):5544–50. 10.1016/j.vaccine.2018.05.119. 10.1016/j.vaccine.2018.05.119 29880241

[14] Baxter R, Bakshi N, Fireman B, Lewis E, Ray P, Vellozzi C (2013). Lack of association of Guillain-Barré syndrome with vaccinations. Clin Infect Dis.

[15] Vellozzi C, Iqbal S, Broder K (2014). Guillain-Barre syndrome, influenza, and influenza vaccination: the epidemiologic evidence. Clin Infect Dis.

[16] Lehmann HC, Hartung H, Kieseier BC, Hughes RA (2010). Guillain-Barré syndrome after exposure to influenza virus. Lancet Infect Dis.

[17] VAERS DATA USE GUIDE. 2017.https://vaers.hhs.gov/docs/VAERSDataUseGuide_October2017.pdf.

[18] Yuki N, Hartung H (2012). Guillain-barré syndrome. N Engl J Med.

[19] Estridge R, Iskander M (2015). Understanding guillain-barré syndrome. J Am Acad Phys Assist.

